# The Impact of Emotionally Focused Therapy on Emotional Distress in Infertile Couples

**Published:** 2013-12-22

**Authors:** Marzieh Soltani, Mohammad Reza Shairi, Rasoul Roshan, Changiz Rahimi Rahimi

**Affiliations:** 1 Department of Clinical Psychology, Shahed University, Tehran, Iran; 2 Department of Clinical Psychology, Shiraz University, Shiraz, Iran

**Keywords:** Depression, Anxiety, Stress, Infertility

## Abstract

**Background:**

The present study investigated the effect of emotionally focused therapy (EFT) on factors contributing to emotional distress among infertile couples.

**Materials and Methods:**

In this semi-experimental study, the subjects consisted of 12 Iranian couples: six infertile men and six infertile women. They were assessed as depressed, anxious and stressful individuals using depression, anxiety and stress scale (DASS). The subjects were randomly divided into control and experimental groups. The experimental group with six couples (i.e. three infertile men and three infertile women) received EFT, while the control group with similar number of couples (i.e. three infertile men and three infertile women) was deprived of the treatment.

**Results:**

There were no significant differences between the two groups regarding job, educational level, income, age, marriage and infertility duration. The pre- and post-test comparisons of DASS subscales showed that level of depression, anxiety and stress among couples with EFT instruction was significantly less than those without such in- structions (p<0.0001).

**Conclusion:**

Emotionally focused therapy could reduce the rate of depression, anxiety and stress in infertile couples, regardless of the man or woman as the cause of infertility.

## Introduction

The need for having children is seen in most couples, while fulfilling such a need is related to their ability of fertility. Infertility means inability of becoming pregnant after one year of sexual intercourse without use of contraceptives (1). The prevalence rate of infertility in different countries varies from 5 to 30 percent. About 20% of couples in developed countries are infertile (2). In Iran, approximately, two million couples are infertile (3).

Infertile individuals experience a strong psychological stress. This stress could affect the relationship between man and woman (4). The infertile individuals experience the symptoms of depression, grief, loss of control and high levels of anxiety (5). In a very recent study, the main emotional disorders of 500 couples from different socio-economic milieu were shock, anxiety, depression, marital disharmony, anger, feelings of guilt, frustration and sense of failure. The infertile couples had, in all mentioned factors, poor well-being than normal couples, so they were susceptible to more physical and psychological stressors (6). Some studies reported that the level of depression and anxiety in infertile people is equal to cancer patients (7). A new study reported depression in 57% and anxiety in 67.2% of infertile women (8).

There are different reasons for psychological problems in infertile people. In societies with cultural norms considering high value for the role of woman as a mother, the consequences of infertility are more severe, and some results such as instability of common life, home violence and loneliness have been observed (9). Different methods of fertility treatment also cause the symptoms of anxiety and depression in 10 to 50% of women (10, 11). After the unsuccessful treatment of zygotic embryos in vitro fertilization (IVF), 25% of women become depressed (11-13). In addition, psychological factors can play a main role in inducing infertility, whereas these factors can be regarded as the consequence of infertility, as well (14). The psychological crisis and the concerns caused by infertility have a direct effect on physiological functions of body and ultimately, a negative influence on infertility (4). The relaxed and healthy individuals experience less psychological tensions, and that consequently increases the possibility of their fertility (15).

The reported evidences about the tensions resulted in infertility have suggested that fertility treatment should be followed by psychological treatment (16, 17). One study (18) found that psychological interventions improved some patients’ chances of becoming pregnant. Although, Boivin (19) reported that group interventions using educational and skills training classes were more effective than methods applying emotional expression and/or discussion about thoughts and feelings related to infertility. One of the psychological treatments which is in line with needs of such couples is Emotionally Focused Therapy (EFT). Infertility, as a source of tension, interferes with emotions (20). In EFT, emotions have central role in couple’s interactions. This approach encourages individuals to talk about their emotions, to discuss about the related subject tin the therapy sessions (21) and to emphasize the restructuring of emotions to secure attachment bonds between partners. Emotional responses can lead to the fulfillment of the person’s needs, and accordingly, improvement of one’s awareness toward emotions is the most fundamental goal of such treatment. Emotion is seen as target and agent of change (22). Harway (23) in a review of some couples therapies (24-27) concluded that behavioral marital treatment and EFT had powerful theoretical basis, and were experimentally valid.

Although both men and women need psychological treatments, depression and anxiety disorders are highly prevalent among women visiting an assisted reproduction clinic for a new course of the treatment (28). Treatment of infertility is a severe stressors process, and many women undergoing fertility treatment experience significant emotional distress (29).

Some studies have investigated infertility and the effectiveness of EFT on couple therapy among Iranian populations. A study on 150 Iranian infertile women showed that infertility, accompanied by numerous psychological and social problems, could affect personal, social and marital relationships, resulting in mental instability and divorce (30). The effectiveness of EFT on marital adjustment in an Iranian sample was investigated (31). EFT resulted in increasing positive feelings of couples toward each other and marital adjustment. Studying the effects of EFT or attachment styles of Iranian couples showed that EFT significantly decreased anxiety and avoidance of couples, while improving the couples’ attachment style (32). The effectiveness of EFT on treatment of other psychological disorders in Iranian couples has already been reported (33, 34).

Because infertility implies strong psychological stress on the individuals, and that influences their relationships with their partners, the purpose of this study was to investigate the application of psychological methods, as complementary treatments, on Iranian infertile couples in order to decrease depression, anxiety and stress.

## Materials and Methods

### Research method

The present research was a semi-experimental study including the experimental and the control groups, and on the basis of pre-and post-test comparison of data. The study was approved by the Ethics Committee of the Shahed University.

### Participants

Participants were recruited from Navid Institute, a well-known fertility center, in Tehran, Iran, using convenience sampling method. This institute was chosen as a main center due to large number of attendances. The couples were diagnosed as infertile by an experienced infertility specialist. The depression, anxiety and stress scale (DASS, 35), a self-report questionnaire, was completed by all participants. Patients were also visited by an experienced clinical psychologist. Those subjects with a history of alcohol and substance abuse, brain damage and any other psychiatric disorders, as measured by DSM-IV-R, were excluded from the study. The desired sample was selected from the infertile couples who, according to DASS, had severe rates (95-98) of depression, anxiety and stress symptoms. The total number of the sample was 63 couples among whom 12 couples with high levels of depression, anxiety and stress were selected (six couples with infertile men and sixcouples with infertile women). Then, the selected couples were randomly divided into the control (n=6 couples) and the experimental groups (n=6 couples). All subjects participated in the study, voluntarily. They declared their satisfaction to participate in the sessions on the basis of a satisfaction form. They also signed a consent form. All respondents answered the questions on demographic issues like gender, age, and educational level.

### Instruments

#### Socio-demographic characteristics questionnaire

In order to obtain the necessary information from the couples, a questionnaire with the following parts was designed: name, name of his/ her spouse, age, educational level, job, income, sexuality, cause of infertility, duration of infertility, number of surgical operations, date of the last surgery, history of participating in any other psychotherapy and infertility counseling sessions, and history of chronic mental and physical illnesses.

#### Depression, anxiety and stress scale

DASS was developed by Lovibond S.H and Lovibond P.F (35) to measure the symptoms of anxiety and depression. This questionnaire consists of 42 items related to the symptoms, liked epression, anxiety and stress. The depression subscale is composed of items measuring patients’ features, like an hedonia, sadness, life futility, worthlessness, hopelessness, unable to become enthusiastic, difficult to work up initiative, lack of energy, lack of self-confidence and nothing to look forward. Anxiety subscale includes items evaluating physiological arousal, phobias and situational anxiety. Stress subscale includes items, such as difficulty in achieving relaxation, state of nervous tension, agitation, overreaction to situations, irritability and restlessness. After reading each item, subjects should rate the severity/frequency of the symptom during the week before in a 4 degree scale (from 0 to 3). Each subscale of depression, anxiety and stress has 14 items, and participant’s score in each subscale is obtained by the sum of all items related to a subscale. In a study with a non-clinical population, the internal consistency of depression, anxiety and stress subscales were, respectively, 0.91, 0.84 and 0.90 (19), while in a study with a clinical population, the internal consistency of these subscales were, respectively, 0.96, 0.93 and 0.89 (36). The re-test coefficients of the three subscales of DASS in a sample of 20 patients with a 2-week interval were 0.71 to 0.81. The 3-factor structure 0f DASS has been approved in different studies. The reliabilities of DASS on the basis of Cronbach alpha, in a study done in Iran (37), were 0.93 for subscales depression, 0.85 for anxiety and 0.87 for stress. Criteria validity of this test showed that correlation coefficient between the scores of depression subscale and Beck depression score was 0.85, anxiety subscale and Zung anxiety scale was 0.83, as well as stress subscale and students stress scale was 0.76. All these findings were significant (p<.01). DASS is interpreted using cut-off scores. Lovibond S.H and Lovibond P.F (35) classified the subjects as normal (0-78), mildly disturbed (78- 87), moderately disturbed (87-95), severely disturbed (95-98) and extremely severely disturbed (98-100). In the present study, subjects completed a Persian version of DASS-42 translated by Afzali et al. (37).

### Emotionally focused therapy program

#### Intervention program

Emotionally Focused Couple therapy program was applied on the basis of Johnson model (50) with the following characteristics: i. first session: Assessment, ii.second session: discrete individual session with each of the couples, iii. third session: identifying the communicative patterns, iv. fourth session: reconstruction of couples’ bond, v. fifth session: deepening affective conflicts of couples on the basis of attachment needs, vi. sixth session: expanding self in relation with others, vii. seventh session: activation, viii. eighth session: finding new solutions for old problems, ix. ninth session: the use of treatment benefits in daily life, and x. tenth session: ending. The content of each session appears with more details in table 1.

**Table 1:** Emotionally Focused Therapy for Couples


Goals	Sessions

Getting familiar with the couple, creating a collaborative therapeutic alliance with them, exploring their interactional potentials and assessing their attachment problems	1
Arranging individual meetings with each of the partners to get information that cannot be obtained if they attend jointly	2
Identifying interaction patterns and the emotional responses shaping such patterns	3
Reconstruction and development of couples' bond	4
Deepening emotional interactions between couples on the basis of attachment needs	5
Expanding self in relation to others	6
Reconstructing interactions, changing the events, as well as clarifying the needs and requests of couples	7
Seeking new solutions to old problems	8
Adopting therapeutic achievements for daily life and involving the couples in friendly relations	9
Facilitating the closure of meetings, while identifying differences between previously held negative interactions, and the newly administered constructive meetings	10


#### Intervention proceeding

In this part, first Session of therapy about assessment is described in more details:

In this session, the couples introduced themselves, individually. The therapist also introduced himself, and then talked about his intervention program. The therapist then explained the consultation process and let the couple know that he understood their aims and needs. He tried to convince them that he was ready to help them. He asked about their reasons for seeking therapy. Each patient was asked to talk individually about his/her problem. The therapist paid special attention to the patients’ initial emotional reactions like crying, blushing, emotional imbalances, etc. He listened to their life story, encouraged them to say more, paid attention to negative/harmful views, organized them as part of a negative cycle, as well as carefully observed their behavior and the way they responded to each other, including where they sat. Attention to the reactions shown by each one of the couple toward the other and the type of emotional, cognitive, behavioral, and interpersonal interactions that they manifested, in response to questions raised, could help to find suitable solutions for their problems. The therapist continued with structured-questions, and directed the session toward their emotional bonds. The therapist then developed his own hypothesis on the main obstacles damaging friendly ties and accordingly resulting in emotional challenges between the couples. He then analyzed the interactions in terms of emotional, cognitive, behavioral and interpersonal relations of the couples, in an attempt to formulate a proper interaction pattern.

#### Procedure

Subjects completed the socio-demographic characteristics questionnaire and DASS in Navid Institute. After completing the questionnaires, 12 couples who had high scores on DASS were selected and randomly assigned as experimental and control groups. Six couples went through couple therapy sessions, while the other six couples, as control group, just waited to be treated. After ending the sessions, both groups again completed the DASS. The therapy sessions were carried out by the first author of the article at the family health clinic of Shahed university under direct supervision of professors of clinical psychology. In line with Johnson’s Pattern (2005), the session lasted for 10 weeks, with one meeting per week.

#### Data analysis

The socio-demographic characteristics of two groups were compared using χ^2^ and Mann-Whitney U test. Descriptive statistics and Mann-Whitney U test were adopted in order to compare depression, anxiety and stress variables in both experimental and control groups, based on score differences between the two groups in the pre and post test results.

## Results

Comparison of socio-demographic characteristics of experimental and control groups using Mann- Whitney U test (Table 2) and χ^2^ (Table 3) showed no significant difference between the two groups regarding variables such as job (p<0.87; χ^2^=0.28), education (p<0.38; χ^2^=1.92), income (p<0.81; χ^2^=0.94), age (p<0.27; Z=1.1), marriage duration (p<0.24; Z= -1.2) and infertility duration (p<0.41; Z=0.83).

**Table 2:** Comparing socio-demographic characteristics of experimental and control groups using Mann-Whitney U Test


Variables	Groups	N	Mean rank	Sum of ranks	z	p

**Age**	Experimental G.	12	10.92	131	-1.10	0<271
	Control G.	12	14.08	169		
**Marriage duration**	.Experimental G	12	10.83	130	-1.17	0<241
	Control G.	12	14.17	170		
**Infertility duration**	.Experimental G	12	11.33	136	-0.82	0<408
	Control G.	12	13.67	164		


**Table 3:** Comparing socio-demographic characteristics of experimental (n=12) and control groups (n=12) using X2 test (12 male, 12 female)


Variables		Experimentalgroup		Control group		Total		df	X^2^	p

N	%	N	%	N	%			
**Education(number of years)**	< 12	5	41.7	4	33.33	9	37.5	2	1.92	0.381
	**12-16**	4	33.33	7	58.3	11	45.8			
	**> 16**	3	25	1	8.3	4	16.7			
**Job**	Housewife	3	25	3	25	6	25	2	0.27	0.871
	**G.employee^1^**	6	50	7	58.3	13	54.2			
	**S.employed^2^**	3	25	2	16.7	5	20.8			
**Income**	No	3	25	2	16.7	5	20.8	3	0.94	0.815
	**Low**	1	8.3	2	16.7	3	12.5			
	**Middle**	7	58.3	6	50	13	54.2			
	**High**	1	8.3	2	16.7	3	12.5			


The findings related to descriptive indexes of pre-test, post-test and participants’ score showing the significant differences in depression, anxiety and stress variables are presented in table 4.

The results related to the comparison of experimental and control groups for the variables of depression, anxiety and stress using Mann- Whitney U Test, shown in table 5, indicated that the acquired Z is, respectively, -3.58, -3.49 and -4.18, which is statistically significant as compared to the critical amounts at alpha level of p<0.0001. This indicates that EFT decreased the rate of depression, anxiety and stress of infertile couples in experimental group in comparison with the control group.

**Table 4:** Means and standard deviations related to pre-test, post-test in depression, anxiety and stress variables of experimental and control groups


Variables	Groups	Pre-test		Post-test		Mean differences	
		M	SD	M	SD	M	SD

**Depression**		Experimental G.	11.75	2.56	7.91	1.67	3.83	2.03
	Control G.	11	2.55	11.25	3.46	-0.25	1.6
**Anxiety**		Experimental G.	14.8	5.53	8.08	2.99	6.0	3.01
	Control G.	12.75	2.8	13.25	4.0	-.0.5	3.37
**Stress**		Experimental G.	17.08	5.79	10.16	3.04	6.91	3.7
	Control G.	16.75	4.43	18.5	4.83	-1.75	1.05


**Table 5:** Comparison of depression, anxiety and stress rates according to pre-test and post-test scores of experimental and control groups using Mann-Whitney U Test


Variables	Groups	N	Mean Rank	Sum of Ranks	z	p

**Depression**	Experimental G.	12	17.58	211	-3.58	0<0001
	Control G.	12	7.42	89		
**Anxiety**	.Experimental G	12	17.50	210	-3.49	0<0001
	Control G.	12	7.5	90		
**Stress**	.Experimental G	12	18.5	222	-4.18	0<0001
	Control G.	12	6.5	78		


## Discussion

Infertility usually causes numerous emotional reactions like depression, anxiety and stress. The effectiveness of EFT in order to reduce the rate of negative emotions of infertile couples in different cultures and societies has been reported. Meanwhile, some studies have reported the effectiveness of EFT in treating other problems in therapy sessions arranged for families and couples in Iran. The present study investigated the effect of EFT on decreasing negative emotions in Iranian infertile couples. The findings indicated that EFT could reduce the rate of depression, anxiety and stress in this group.

These findings are consistent with the results of some studies in different cultures (38-42) which have referred to the effectiveness of EFT in reducing emotional discomforts. According to other studies, compared to pharmacotherapy, EFT was more effective in reducing depression among couples (43), especially for couples with anxious wife (44). The findings of present study are in line with the findings of some studies in Iran, according to which EFT elevated the couples’ positive feelings toward each other resulting in marital adjustment (23); in addition, it significantly decreased anxiety and avoidance of couples, while improving their attachment style (24).

It is important to consider that such positive effects may be rooted in the characteristics of EFT because the program only focuses on emotions, and the aim is to complete catharsis of each couple’s emotions. The therapist should discuss issues such as discovering attachment insecurity and couples’ fears, clarifying the emotional key response, developing emotional experiences of couples, deepening individual relationship with emotional experience, deepening emotional struggles, identifying the basic fears and ultimately inducing secure attachment in the sessions; this can be one of the reasons for the effectiveness of EFT pattern among these couples. Therefore, we selected couples who had an emotional disorder (depression, anxiety and stress) and a specific problem (infertility) because emotional problems such as depression, anxiety and stress are very familiar experiences for couples with infertility. The main focus was, therefore, the problems of infertile couples and the effectiveness RFT pattern in treating such groups.

## Conclusion

Infertile couples experience negative emotions like anxiety, stress, and especially depression which could result in social and marital problems. Fertility problem involving man or woman is a problematic issue in Iranian society. It was revealed that EFT can reduce the rate of depression, anxiety and stress in Iranian infertile couples. Therefore, due to its significant effect in preventing severe psychological problems, EFT is recommended as a remedy for reducing infertility problems. The infertile persons are encouraged to use psychological services. Nonetheless, it is necessary to mention that this study used just a limited number of subjects with infertility problems. Also, the study did not investigate the effect of the participants’ sex, although it may have a share in inducing emotional problems. As well as, the cause of infertility can play an important role in psychological disorders of patients, but it was not investigated in this research. The future studies can account for such limitations. Despite such limitations, the current study proved to be a success; however, the question still waiting to be answered is whether future studies would supports the findings of the current work. Undoubtedly, execution of a significant number of studies on intensive emotional therapy, in different groups of patients, under various socio-cultural conditions could indicate if there is sufficient evidence to remain positive for similar interventions in future.
